# Absence of Correlation between Chimeric RNA and Aging

**DOI:** 10.3390/genes8120386

**Published:** 2017-12-14

**Authors:** Reyna Huang, Shailesh Kumar, Hui Li

**Affiliations:** 1 Department of Pathology, School of Medicine, University of Virginia, Charlottesville, VA 22908, USA; rlh3xt@virginia.edu (R.H.); shailesh.97@gmail.com (S.K.); 2 National Institute of Plant Genome Research (NIPGR), New Delhi 110067, India; 3 Department of Biochemistry and Molecular Genetics, School of Medicine, University of Virginia, Charlottesville, VA 22908, USA

**Keywords:** chimeric RNA, aging, *cis*-splicing of adjacent genes, *trans*-splicing

## Abstract

Chimeric RNAs have been recognized as a phenomenon not unique to cancer cells. They also exist in normal physiology. Aging is often characterized by deregulation of molecular and cellular mechanisms, including loss of heterochromatin, increased transcriptional noise, less tight control on alternative splicing, and more stress-induced changes. It is thus assumed that chimeric RNAs are more abundant in older people. In this study, we conducted a preliminary investigation to identify any chimeric RNAs with age-based trends in their expression levels in blood samples. A chimeric RNA candidate list generated by bioinformatic analysis indicated the possibility of both negative and positive trends in the expression of chimeric RNAs. Out of this candidate list, five novel chimeric RNAs were successfully amplified in multiple blood samples and then sequenced. Although primary smaller sample sizes displayed some weak trends with respect to age, analysis of quantitative PCR data from larger sample sizes showed essentially no relationship between expression levels and age. Altogether, these results indicate that, contradictory to the common assumption, chimeric RNAs as a group are not all higher in older individuals and that placing chimeric RNAs in the context of aging will be a much more complex task than initially anticipated.

## 1. Introduction

Traditionally, it was thought that chimeric RNA events were exclusively characteristic of the cells of neoplasms [1], but evidence has shown the presence of chimeric RNAs in various physiologically normal tissue [2,3]. Furthermore, because of their presence in cancers, fusion RNAs were once thought to be the sole result of chromosomal translocations [4], but other work has recently shown that they can exist without DNA arrangement and rather through two mechanisms called *cis*-splicing of adjacent genes (*cis*-SAGe) [5,6,7,8], and *trans*-splicing [9]. *Cis*-SAGe involves the splicing of a singular pre-mRNA molecule that results from passing through the termination site between two adjacent genes. On the other hand, *trans*-splicing involves the splicing that connects separate transcripts.

Much research has been invested into elucidating the biology of aging in recent years. Many have tried to identify characteristics and understand mechanisms that contribute to aging due to its role as a major risk factor in many chronic diseases [10], such as cardiovascular disease and cancer, that rank amongst the top causes of death in developed nations. Most indisputable is the genome damage that accompanies aging in organisms, but just how that damage affects cell and tissue function and vitality is more complex.

Previous studies have found pronounced heterochromatin loss in individuals with progeroid syndromes, which is characterized by accelerated aging in affected children [11], and similarly, non-neuronal cell types in *Caenorhabditis elegans* have shown progressive loss of heterochromatin in an age-dependent manner [12,13]. Such a loss of heterochromatin causes the expression of genes that are normally repressed and, therefore, aberrant transcription that may be associated with a variety of RNA classes. Another aspect of aging is transcriptional noise, which is the differential gene expression of cells in an isogenic population, and this phenomenon is correlated with genome damage. This transcriptional noise has also been implicated in reduced organismal fitness [14], which is essentially what is recognized as aging. Similarly, certain transcription levels of protein isoforms have been shown to change with age, and specifically, some of these isoforms cause the deregulation of mechanisms in alternative splicing [15], which would directly increase the occurrence of abnormal splicing products. Recent work has also taken a look into the genetic level of stress, and the results support the increase of read-through transcripts under many types of biological stress, including osmotic-induced stress, heat shock, oxidative stress, and viral infection [16].

We therefore hypothesize that frequency of chimeric RNA events will have a positive correlation with age due to expected age-dependent deregulation of transcription machinery, particularly in the case of *cis*-SAGe. With our study, we aimed to confirm the existence of chimeric RNA candidates and elucidate the presence of trends, if any, with respect to age.

## 2. Materials and Methods

### 2.1. RNA-Seq and Bioinformatics Analyses

The Genotype-Tissue Expression (GTEx) raw RNA-Seq data was downloaded. Next Generation Sequencing Quality Control toolkit (http://www.nipgr.res.in/ngsqctoolkit.html) was used for filtering off low-quality reads. Paired end sequencing reads were mapped to Human genome version hg19 and analyzed using software tool, EricScript to identify candidate fusion RNAs [17]. Fusions with Ericscore less than 0.5 were filtered off. The occurrence and frequencies of candidate fusion RNAs were then correlated to gender, age, race, ethnicity, height, weight, and Body Mass Index (BMI).

### 2.2. Sample Collection

The use of human clinical samples was approved by the IRB committee of the University of Virginia (#13310, 8/28/2017). Blood samples were obtained from the Department of Pathology at the University of Virginia. All of the samples were de-identified.

### 2.3. RNA Extraction

The RNA was extracted with TRIzol reagent (Invitrogen, Waltham, MA, USA) following the manufacturer’s instruction. Extracted RNA was then treated with DNaseI and reverse-transcribed by random primers using AMV (New England Biolabs, Ipswich, MA, USA). More detailed procedures have been previously described [18].

### 2.4. RT-PCR and Sanger Sequencing

Specific primer pairs that were designed using Primer3 (Whitehead Institute for Biomedical Research, Cambridge, MA, USA) were utilized in Reverse Transcription Polymerase Chain Reaction (RT-PCR) to confirm candidates on the RNA level with quantitative RT-PCR. Amplification products were separated through gel electrophoresis, and proper size product bands were purified and sent for Sanger sequencing. More detailed procedures have been previously described [18,19].

### 2.5. Statistical Analyses

All quantitative RT-PCR amplification data for the samples were normalized to their *GAPDH* values to control for general transcription levels between samples. The correlation between normalized fusion RNA levels and age of the sample were calculated using the Pearson correlation method, with *R*-values.

## 3. Results

### 3.1. Bioinformatic Analysis of GTEx Data

**Figure 1:**
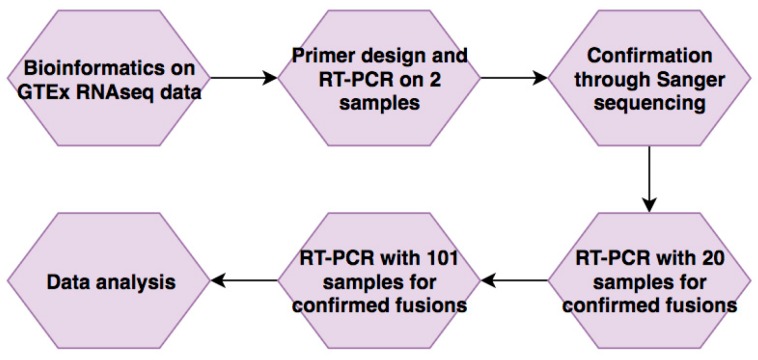
The flow of the study. A list of candidate fusion RNAs that have correlations with aging was generated through bioinformatic analyses on Genotype-Tissue Expression (GTEx) RNA-Seq data. They were then validated using Reverse Transcription Polymerase Chain Reaction (RT-PCR) and Sanger sequencing. Five fusions passed this step and were then evaluated in 20 samples, then extended to 101 samples. Their expression levels were then correlated with donors’ age.

Starting with bioinformatics, our work followed a pipeline through which candidates were identified and then narrowed down through confirmation before testing in larger sample sizes for their expression (Figure 1). We wanted to avoid the effect of neoplasm, so we started with a data set collected from non-cancer samples. The Genotype-Tissue Expression (GTEx) project provides an ideal resource to study non-cancer associated fusions [20], in that the samples were procured from non-cancer patients, and the paired-end RNA-Sequencing datasets are publically available. We mined 426 GTEx whole blood RNA-Seq data to identify candidate fusion RNAs. Software Ericscript [17] was used to identify fusion RNAs. Fusion RNAs with Ericscore above 0.5 were further analyzed. *p*-Values were then calculated to determine correlation with the samples’ various characteristics, including gender, age, race, ethnicity, height, weight, and BMI with the frequency with which they were found. Age-correlation was investigated through a x^2^ test comparing four different age groups that split the sample numbers evenly. A list of 65 top candidates was then generated by compiling the most significant *p*-values with respect to age. When we examined other variables, no significant *p*-values were found. Contrary to our initial expectations, some of these candidates may also decrease in frequency with age (Table 1).

### 3.2. Confirmation of Five Candidates through RT-PCR and Sanger Sequencing

**Figure 2:**
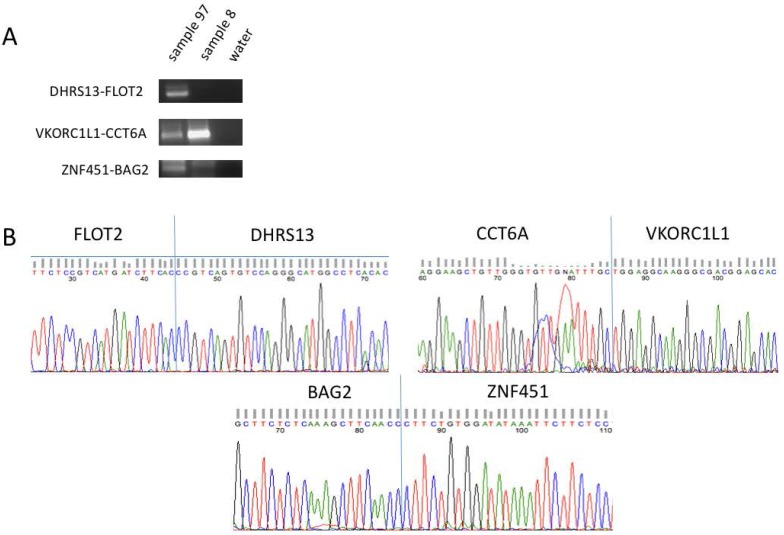
Confirmation of fusion RNAs. (**A**) Representative examples of fusion RNAs amplified by RT-PCR and separated by gel electrophoresis before being purified and processed for Sanger sequencing. The size of the fusion RNAs fall within 100–300 bp. (**B**) Sanger sequencing data highlighting the junction sequence for the same representative fusions.

We designed pairs of specific primers to amplify the candidate fusion RNAs. Eleven of the 65 had no primers successfully designed for them due to their highly repetitive and nonspecific sequences. For the remaining 54, we designed primers and performed quantitative PCR (qPCR). To quickly survey through this list of candidates for age-biased fusions, we ran one sample extracted from an 80-year-old, and one sample from a 30-year-old. Water was included as the negative control. After running the amplified products on a gel, any clear bands of correct product size that also presented themselves in the given expected trend from the bioinformatics results were purified and processed for Sanger sequencing (example in Figure 2A). Therefore, while some bands were of the correct product size, they were not investigated further if they did not show the same trend as predicted. In addition, there may be candidates that could not be amplified in the particularly small sample size.

From this step, five separate fusion RNAs were confirmed through Sanger sequencing, including *ATXN1L-IST1*, *DHRS13-FLOT2*, *LRP10-REM2*, *VKORC1L1-CCT6A*, and *ZNF451-BAG2* (example in Figure 2B). Two separate bands were purified and sequenced for *LRP10-REM2*, confirming two different forms of the fusion, one between the 6th exon of *LRP10* and the 2nd exon of *REM2* (e6e2), the other being the 7th exon of *LRP10* and the 2nd exon of *REM2* (e7e2), representing the original expected junction site. All five of these fusions were composed of genes on the same chromosome, and in some cases, immediate neighboring genes, thus candidates for *cis*-SAGe (*DHRS13-FLOT2*, *LRP10-REM2*, and *ZNF451-BAG2*).

### 3.3. Investigation of the Correlation between the Five Candidates and Age of the Donors

**Figure 3:**
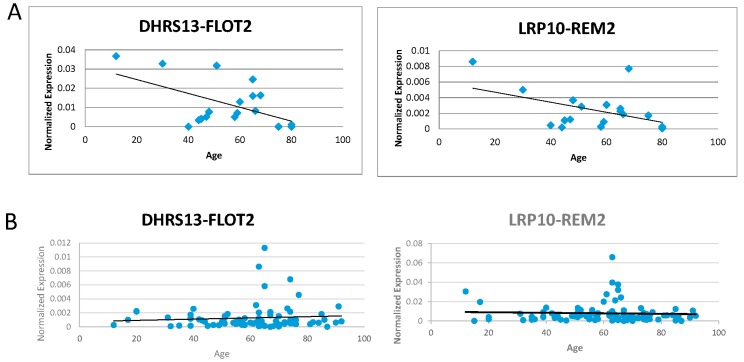
Correlation of fusion RNAs with aging. (**A**) *DHRS13-FLOT2* and *LRP10-REM2* (both forms) normalized expression levels graphed against age with 20 samples. (**B**) *DHRS13-FLOT2* and *LRP10-REM2* (both forms) normalized expression levels graphed against age with 101 samples.

We then attempted to amplify these five confirmed fusions on complementary DNAs (cDNAs) extracted from 20 different blood samples. Correlation was calculated between the fusion RNAs expression for each sample and the age of the patient that each sample was attributed. *ATXN1L-IST1* showed a moderate negative correlation with age, while *DHRS13-FLOT2* and *LRP10-REM2* had stronger negative correlations with age. Data showed that *VKORC1L1-CCT6A* had a weak positive correlation in amplification with age, but nearly no correlation could be made between *ZNF451-BAG2* and age (examples in Figure 3A) (Table 2).

We then measured the fusion RNAs using a group of 101 cDNA samples to see if similar correlations could be found in a larger sample size (Table S1). Most trends either flipped or decreased in their absolute *R*-value, or both. *ATXN1L-IST1*, *DHRS13-FLOT2*, and *ZNF451-BAG2* went from negative trends to only marginally positive trends. *VKORC1L1-CCT6A* went from a positive trend to negative. The correlation of *LRP10-REM2* did not change in direction, but its absolute *R*-value decreased from −0.45383219 to −0.0464758 (examples in Figure 3B) (Table 2). No correlation of the expression of *GAPDH* with age was observed (Figure S1).

### 3.4. Alternate Forms of LRP10-REM2 Correlate in Expression with Each Other, But Had No Correlation with Age

**Figure 4:**
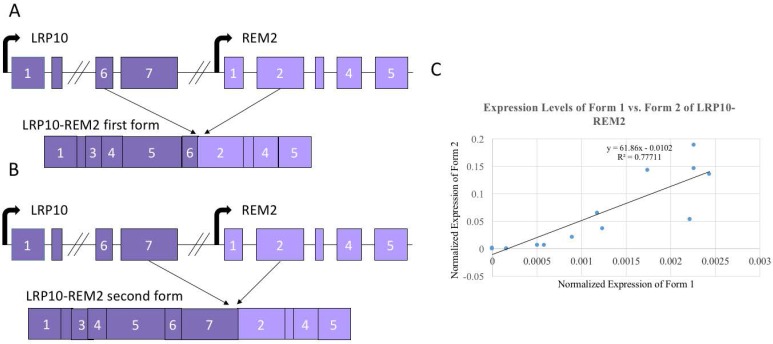
Two isoforms of *LRP10-REM2.* (**A**) Exon diagram of the first form with 6th *LRP10* exon joining to the 2nd *REM2* exon (e6e2) of *LRP10-REM2*. (**B**) Exon diagram of the second form with 7th *LRP10* exon joining to the 2nd *REM2* exon (e7e2) of *LRP10-REM2*. (**C**) Correlation between the expression levels between the two forms of *LRP10-REM2*. Diagrams not to scale with base pair length.

Since *LRP10-REM2* had two forms that were confirmed through Sanger sequencing, we designed assays specifically for each form (Figure 4A,B). To specifically detect the form with the junction between the 7th *LRP10* exon and the 2nd *REM2* exon (e7e2), a new forward primer was designed to be specific to the 7th exon of *LRP10*, which would avoid amplifying the fusion with 6th *LRP10* exon and the 2nd *REM2* exon form (e6e2). Then, to specifically detect the e6e2 form, a Taqman probe annealing to the junction site between the 6th exon of *LRP10* and the 2nd exon of *REM2* was designed. Twenty samples were used first to compare the two forms. A strong positive correlation with the expression levels of the two forms (*R*^2^ = 0.77711) was found (Figure 4C). Because of this, it was determined that using the set of primers that amplify both forms simultaneously would be proper to investigate the correlation of both fusions and age. As shown in Figure 4, no significant correlation was seen for *LRP10-REM2*.

### 3.5. Alternate Analysis of the Data with Age Buckets also Failed to Show a Prominent Trend

**Figure 5:**
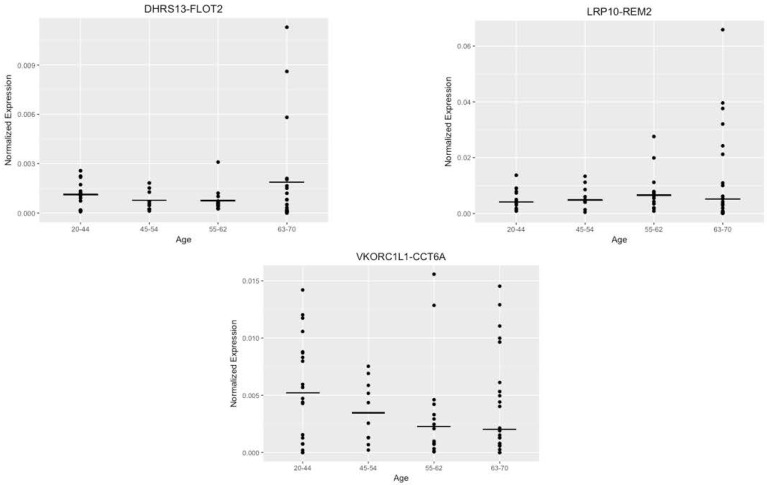
Alternate analysis of the fusion expression with age buckets. Dot plots representing distribution of normalized expression by age groups (20–44, 45–54, 55–62, 63–70) for *DHRS13-FLOT2*, *LRP10-REM2* (both forms), and *VKORC1L1-CCT6A.*

After attempting to correlate the normalized expression levels with age as a continuous variable, we created age buckets identical to those used in the bioinformatics stage and compared the average expression levels of the samples that fell within these buckets to each other (Figure 5). This was an attempt to confirm any significance detected in the bioinformatics stage of the pipeline. The generated graphs exhibited no particular trend for any of the fusions except for *VKORC1L1-CCT6A*, but this trend was negative. This contrasts with the bioinformatics analysis, which predicted a positive trend with age.

## 4. Discussion

In this study, we failed to establish with any strongly supporting evidence the existence of any age-biased trends in the expression of fusion RNAs. Even though certain fusion RNAs have been previously shown to be accurate biomarkers of certain diseases and these diseases affect a larger proportion of older individuals, no strong correlations could be made with age with the larger sample size.

Interestingly, not all of the fusion candidates uncovered from the RNA-Seq data were found in increasing frequency with age. This already contradicts our hypothesis and many of the aforementioned mechanisms that are associated with aging. This also suggests these candidates or fusion RNAs are not simply the byproducts of dysregulated transcription machinery. What their roles actually are would require further work to elucidate.

The lack of significant age-based trends in the expression levels of these confirmed fusions in a larger sample size, may be partially due to the complexity of aging. Aging carries countless confounding variables that are difficult to control for with de-identified samples. The blood samples used in this study were collected from various patients in the hospital, so profiles of health conditions and demographics varied greatly. Furthermore, due to the de-identification process, it was impossible to know how much healthier one sample was compared to another, which is a relationship that a numerical age may improperly represent. Thus, at this point, we had to confront the concept of difference in years since one’s birth and the current aging state one is in. In future work, it would be necessary to consider just what sample size and what demographics would be sufficient to statistically support bioinformatically predicted trend. 

There are also limitations associated with the use of blood samples for studying age. Previous work has already shown that there is differential expression of fusion RNAs between tissues [21], so contrasting results may have been collected if different tissues were used as samples. Another caveat of using blood can be realized when considering how different tissues relate to aging. Any red blood cell in the human body will stay in circulation for approximately 120 days, meaning that unlike other cell types, such as neurons, a blood cell would not age with an individual through their lifespan. However, the relative availability of blood samples compared to relatively difficult collection of any other tissues from individuals of all ages made it our first choice to probe the question.

## Figures and Tables

**Table:** (**A**)

Candidate	Frequency	Cohort	Gender	Age	Race	Ethnicity	Height	Weight	BMI
*ADCK4-NUMBL*	16	2.4 × 10^−2^	1.90 × 10^−1^	3.60 × 10^−2^	9.39 × 10^−1^	1	9.90 × 10^−2^	4.40 × 10^−1^	3.01 × 10^−1^
*ATXN1L-IST1*	7	1.24 × 10^−1^	1	3.20 × 10^−2^	9.95 × 10^−1^	1	2.14 × 10^−1^	5.10 × 10^−2^	9.40 × 10^−2^
*DHRS13-FLOT2*	22	1.72 × 10^−4^	4.94 × 10^−1^	4.00 × 10^−2^	5.95 × 10^−1^	1	8.20 × 10^−1^	5.44 × 10^−1^	6.31 × 10^−1^
*LRP10-REM2*	31	1.49 × 10^−1^	7.09 × 10^−1^	4.00 × 10^−3^	9.45 × 10^−1^	1	8.96 × 10^−1^	8.77 × 10^−1^	4.73 × 10^−1^
*AMN1-RBM28*	51	1.01 × 10^−5^	6.52 × 10^−1^	2.00 × 10^−2^	5.06 × 10^−1^	7.05 × 10^−1^	5.49 × 10^−1^	1.85 × 10^−1^	3.03 × 10^−1^
*C1orf112-SMARCA4*	23	9.00 × 10^−4^	1	6.00 × 10^−3^	7.54 × 10^−1^	1	9.80 × 10^−1^	9.58 × 10^−1^	5.96 × 10^−1^
*C17orf99-SYNGR2*	7	1.23 × 10^−1^	9.55 × 10^−1^	4.00 × 10^−3^	7.50 × 10^−1^	1	9.54 × 10^−1^	3.79 × 10^−1^	9.30 × 10^−1^
*EIF4A1-SENP3-EIF4A1*	54	2.20 × 10^−5^	5.00 × 10^−3^	4.50 × 10^−2^	2.55 × 10^−1^	1	5.70 × 10^−1^	2.94 × 10^−1^	9.02 × 10^−1^
*ARHGEF39-CCDC107*	59	5.11 × 10^−10^	4.19 × 10^−1^	3.00 × 10^−3^	5.82 × 10^−1^	9.42 × 10^−1^	8.16 × 10^−1^	7.34 × 10^−1^	6.75 × 10^−1^

**Table:** (**B**)

Fusion	Forward Primer	Reverse Primer	Predicted Trend
*ATXN1L-IST1*	AGAGGACAAGAAAGCTGGTCAC	ggctcaaagccagatcttctaa	negative
*DHRS13-FLOT2*	ACCGAATTCAGGCTAAAGTTGA	tgatgtcctgcacattcttacc	positive
*LRP10-REM2*	GCTACAGATCTTACGCCAGGAT	tggccagtcaagttcatctaca	positive
	CTTGCTCCCTCGAACCAAC *		
*VKORC1L1-IST1*	AATCCTGCTCTCCATCTACGC	ttcagcagctctccaatgatta	positive
*ZNF451-BAG2*	TGATAACATGGGTGCCAAAA	tctcaccgtcactgatctgc	negative

**Table 2:** Summary of *R*-values of normalized expression levels correlated with age for confirmed fusions at the 20 sample and 101 sample level.

Fusion	20 Sample *R* Value	101 Sample *R* Value
*ATXN1L-IST1*	−2.36 × 10^−1^	1.08 × 10^−1^
*DHRS13-FLOT2*	−5.25 × 10^−1^	5.25 × 10^−2^
*LRP10-REM2*	−4.54 × 10^−1^	−4.64 × 10^−2^
*VKORC1L1-CCT6A*	2.36 × 10^−1^	−3.04 × 10^−1^
*ZNF451-BAG2*	−8.25 × 10^−2^	3.05 × 10^−2^

## Supplementary Materials

The following are available online at www.mdpi.com/2073-4425/8/12/386/s1. Figure S1: The absence of correlation of *GAPDH* with aging. Table S1: Age of the clinical samples used in Figure 3, Figure 4 and Figure 5 and Figure S1.
