# Supplementary material for: Absence of Correlation between Chimeric RNA and Aging
Source: Genes (Basel). 2017 Dec 14;8(12):386. doi: 10.3390/genes8120386 (PMC5748704; doi:10.3390/genes8120386)
Supplement: Supplementary file 1 [file genes-08-00386-s001.pdf]

Supplementary Materials

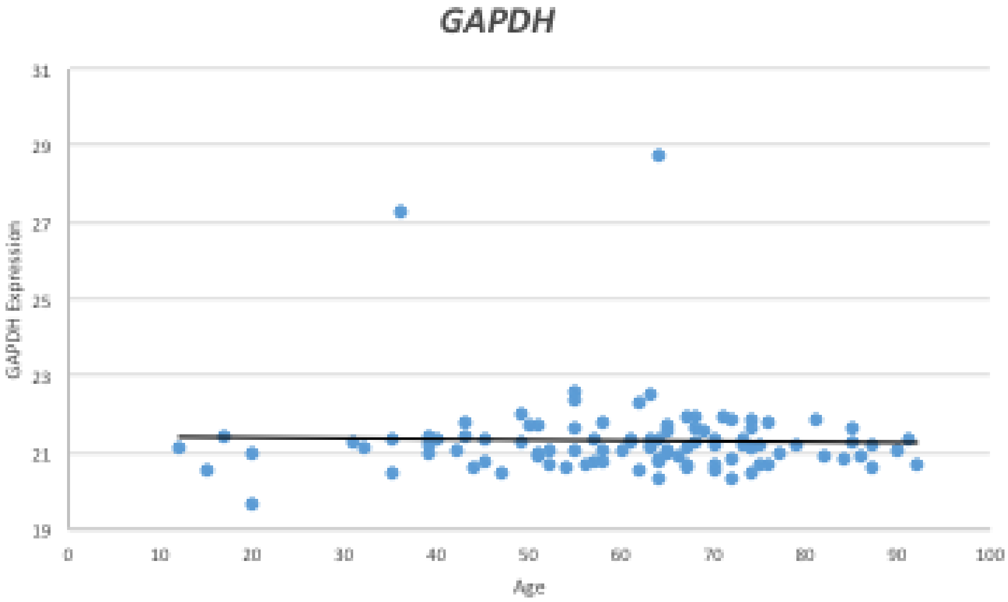

Figure S1. The absence of correlation of GAPDH with aging. *GAPDH* expression levels graphed against age with 101 samples.

Table S1. Age of the clinical samples used in Figures 3–5 and Figure S1.

Sample	Age
1	68
2	64
3	52
4	56
5	51
6	55
7	55
8	67
9	75
10	35
11	36
12	55
13	43
14	49
15	71
16	72
17	64
18	67
19	45
20	73
21	68
22	61
23	69
24	50
25	64
26	72
27	58
28	65
29	67
30	90
31	74
32	57
33	74

Sample	Age
34	42
35	39
36	43
37	40
38	70
39	39
40	73
41	85
42	49
43	68
44	31
45	70
46	87
47	79
48	63
49	63
50	74
51	32
52	65
53	55
54	58
55	52
56	39
57	20
58	77
59	51
60	51
61	66
62	86
63	82
64	64
65	84
66	72

Sample	Age
67	45
68	57
69	64
70	58
71	70
72	75
73	76
74	92
75	67
76	44
77	54
78	87
79	70
80	62
81	15
82	47
83	74
84	35
85	76
86	65
87	62
88	20
89	91
90	85
91	74
92	17
93	44
94	12
95	63
96	63
97	81
98	60
99	65
100	39
101	61
